# Determinants of mental health service use in the national mental health survey of the elderly in Singapore

**DOI:** 10.1186/1745-0179-5-2

**Published:** 2009-01-19

**Authors:** Ma Shwe Zin Nyunt, Peak Chiang Chiam, Ee Heok Kua, Tze Pin Ng

**Affiliations:** 1 Gerontological Research Programme, Faculty of Medicine, National University of Singapore, Singapore; 2 National University of Singapore, Department of Psychological Medicine, Singapore; 3 Institute of Mental Health, Ministry of Health, Singapore

## Abstract

**Background:**

Despite high prevalence of mental health problems, only a minority of elderly people seek treatment. Although need-for-care factors are primary determinants of mental health service use, personal predisposing or enabling factors including health beliefs are important but are not well studied.

**Method:**

In the National Mental Health Survey of Elderly in Singapore, 2003, 1092 older adults aged 60 and above were interviewed for diagnosis of mental disorders (using Geriatric Mental State) and treatment, and their health beliefs about the curability of mental illness, embarrassment and stigma, easiness discussing mental problems, effectiveness and safety of treatment and trust in professionals.

**Results:**

The prevalence of mental disorders was 13%, but only a third of mentally ill respondents had sought treatment. Increased likelihood of seeking treatment was significantly associated with the presence of a mental disorder (OR = 5.27), disability from mental illness (OR = 79.9), and poor or fair self-rated mental health (OR = 2.63), female gender (OR = 2.25), and formal education (OR = 2.40). The likelihood of treatment seeking was lower in those reporting financial limitations for medical care (OR = 0.38), but also higher household income (OR = 0.31). Negative beliefs showed no meaningful associations, but the positive belief that 'to a great extent mental illness can be cured' was associated with increased mental health service use (OR = 6.89). The availability of family caregiver showed a negative association (OR = 0.20).

**Conclusion:**

The determinants of mental health service use in the elderly included primary need factors, and female gender and socioeconomic factors. There was little evidence of influences by negative health beliefs, but a positive health belief that 'mental illness can be cured' is a strongly positive determinant The influence of family members and care-givers on senior's use of mental health service should be further explored.

## Background

Numerous reports have shown that despite a sizeable need for mental health services evidenced by the high prevalence of mental disorders, only a minority of mentally unwell persons receives treatment in the healthcare system [1-5]. The factors determining health service use for mental illness are complex and not fully understood. A body of evidence [6-8] has implicated symptom profile, severity and duration, associated disability and other characteristics of the mental disorder itself in explaining mental health service use. However, such need-for-care factors do not fully determine actual health seeking behavior; there are critical personal, social and cultural factors that are not well studied. Such predisposing and enabling factors include age, sex, race, education, income, social network and support, and health beliefs – which are attitudes, values and knowledge that people have about their health and health services and that influence their perceptions of need and use of health services [9]. They include self-assessment of mental health status and perceived need for help, negative beliefs and attitudes as regards the causes and nature of mental illness, stigma and lack of trust with professionals [4,10-12] difficulty in discussing mental health problems, overestimation of one's coping abilities [13,14] and the perceived or actual reactions of one's family and friends [15]. Few authors have investigated the extent to which personal health beliefs and behavior contribute to the underutilization of mental health services [7].

Using data from a national sample of the Singaporean elderly population in the National Mental Health Survey of Elderly, 2003, we examined mental health service use and the relative extent to which it was determined by need-of-care, sociodemographic, health beliefs, and family and social support factors.

## Methodology

### Study design and subjects

The analysis in this study was based on data collected from the National Mental Health Survey of the Elderly (NMHS-E) [16,17], a population-based survey of a nationally representative sample of older adults, conducted between 15 February 2003 and 30 March 2004. The respondents were a stratified random sample of older adults aged 60 and over. The sample was disproportionately stratified by ethnicity, such that Malays and Indians were oversampled to enhance the statistical precision of ethnic-specific estimates for outcome measures of interest. The final effective sample comprised 48% Chinese, 33% Malays and 19% Indians.

The sampling list of household addresses was generated by the Department of Statistics using pure probability sampling from a national sampling frame of dwellings. In each household, eligible persons who were older adults aged 60 or above and Singapore citizens or permanent residents were identified. Subjects were excluded if they were physically or mentally unable to participate. One person per household was randomly selected. A total of 1092 elderly people participated in the survey (72.4% response rate). Respondents gave written informed consent for the study, which was approved by the Ethics Committee of the Institute of Mental Health.

A multi-ethnic and multi-language team of trained field interviewers interviewed the respondents in their homes. The majority of respondents (87%) gave self-report information; where subjects were unable to do so, proxy reports were obtained from caregivers (13%). Interviews were conducted in English, Mandarin or other Chinese dialects and Malay. Indian respondents were interviewed in either English or Malay, as they were mostly conversant in either language.

### Measurements

#### Diagnosed mental disorders

The diagnosis of a mental disorder in the past 12 months was determined by means of a semi-structured diagnostic interview using the Geriatric Mental State (GMS) instrument, a validated instrument that is widely used to assess psychopathology for older adults in the community [18]. The GMS was translated into Chinese and Malay, and administered by trained psychiatric and community nurses and interviewers at the subjects' homes. The interviewers underwent extensive training in the use of the GMS using training vignettes, completed and co-rated four to six supervised training interviews, and received close supervision and monitoring of their field interviews. The trainers and supervisors were psychiatrists who were trained by developers of the GMS at the Institute of Psychiatry, London (KEH, CPC). Depressive disorders and other psychiatric morbidities were diagnosed by means of a computer-assisted system, the Automated Geriatric Examination for Computer Assisted Taxonomy (AGECAT)[18]. The system provides diagnosis of recent psychiatric disorders based on items related to organic, affective and anxiety syndromes by generating five levels of diagnostic confidence for determining 'caseness'. Diagnostic confidence levels of 3 to 5 ('case') meets criteria for DSM-IV diagnoses of dementia, depression and anxiety and other disorder.

Also the respondents were asked to report whether they had been treated by a doctor for mental or emotional problems and to show all their medications that they had been taking in the past 12 months. Those who reported having been treated by a physician for mental disorder and with positive identifications of psychiatric medications were also considered to have a diagnosis of a mental disorder.

Participants were asked to rate their mental health status as "poor, fair, good, very good or excellent" and how long they had been having a "severe mental or emotional health problem that interferes with your life and activities".

*Mental health service utilization *was determined by the response to a question, 'In the past 12 months, have you sought professional help or treatment for problems with mental or emotional problems, from a general practitioner, psychiatrist, psychologist, psychiatric nurse, social worker and mental health counselor, and others (please specify)."

*Sociodemographic data *were collected and categorized as gender, age (< 75 yrs & ≥ 75 yrs), ethnicities (Chinese, Malay, Indian), languages used (English, Chinese, Malay), level of education (no formal education or at least primary educational level), marital status (whether single, divorced and widowed), and living arrangement (whether living alone), employment status (active paid employment versus unemployed or retired versus housewives), and monthly total household income (low: < S$1,000, moderate: S$1000 – 2999 and high: > S$3,000), and response to a question whether the subject had financial resource limitation for medical care (not at all, to some extent, to a great extent).

The respondents' health beliefs that included perceptions or views about mental illness, professional care and medications and one's own problem solving skills were assessed by their responses on 3-point (1 = not at all, 2 = to some extent, 3 = to a great extent) or 5-point Likert scales (1 = strongly disagree to 5 = strongly agree) to statements about the curability of mental illness, embarrassment and stigma, easiness discussing mental problems, effectiveness and safety of treatment and trust in professionals (See Table): "My problem can be overcome without help from a medical professional", "Drugs prescribed by doctor often do more harm than good". "Mental health professionals can do very little to help people with mental health or emotional problems", "Are you embarrassed or ashamed about mental ill health or emotional problems?", "Are your religious or spiritual beliefs a source of support and comfort to you?", "Do you believe that mental illnesses can be cured?" Respondents were given to understand that mental health professionals referred to formal healthcare providers in the medical care system.

Other variables included whether they had a regular source of medical care, and their preference to seek a health professional for help for serious mental problem, and the extent to which they were limited by financial resources to pay for needed medical services,

### Statistical analysis

Bivariate associations of individual need, enabling and predisposing variables with mental health service use were assessed by χ^2 ^tests for categorical variables. In multivariate logistic regression analyses, all variables were entered as candidate variables into a full model and in a reduced final model from backward elimination procedures (p < 0.10 for removal) of significant variables that simultaneously predicted health service use. We estimated odds ratio (OR) and their 95% confidence intervals (C.I.) of association, controlling for other variables. The data analysis took into account the survey design based on a one-stage pure probability sampling of households with one elderly person from each household. Hence, only weighted analysis using ethnic distribution of older adults in the general population was performed. All analyses were performed using SPSS software version 13.0 (SPSS Inc, Illinois, Il).

## Result

The mean age of the 1092 respondents in the sample was 69.7 (standard deviation 7.4) years; 25% were aged ≥ 75 years; 56.2% were women.

The presence of a mental disorder was determined in a total of 125 respondents, giving a population weighted prevalence estimate of 13.0%. The cases included 101 who were diagnosed from GMS interview (of whom 13 reported past treatment for mental illness) and an additional 23 who were not positively identified by GMS but gave reports of past treatment for metal health problems, evidenced by the use of anti-depressant and other psychiatric drugs. The diagnoses of psychiatric disorders made by GMS included dementia (56.2%), depression (34.3%), mania (4.8%), anxiety (1.9%), and schizophrenia (2.8%).

A total of 74 respondents reported use of health services for mental or emotional problems, giving an overall population weighted estimate of mental health service utilization rate of 7.0%. (Table 1) They comprised 42 (33%) among 125 respondents with a diagnosed mental disorder, and 32 (3.2%) among the remaining 967 respondents without a diagnosed psychiatric disorder. Overall in the elderly population, the majority of users of mental health services were treated by primary care physicians (58.1%), and among respondents with a diagnosed psychiatric disorder, a majority of respondents were treated by psychiatrists (45.2%). Only a minority (16.2%) used multiple sources of service providers, and 13.4% used alternative medicine or religious service providers (clergy, traditional healers and spirit mediums) for help with mental health problems. Among 10 respondents who sought help from clergy, traditional healers and spiritists, 3 of them sought the help of health professionals as well.

**Table 1:** Mental health service utilization in Singapore older adults aged 60 and above (N = 1092)

	General population (N = 1092)		Diagnosed mental disorders (N = 125)	
		
Mental health service utilization	N	%	N	%
Mental health service users	74	6.9 *	42	33.6*
1 source	62	83.8	33	78.6
2 or more sources	12	16.2	9	21.4
				
Health service providers:				
General Practitioners	43	58.1	16	38.1
Psychiatrist	22	29.7	19	45.2
Mental health nurses	2	2.7	2	4.8
Social worker	3	4.0	0	0
Mental health counselor	3	4.0	2	4.8
				
Traditional medicine service providers	3	4.0	3	7.1
				
Religious service providers: clergy, spiritists, mediums	7	9.4	4	9.5

* Population-weighted estimate of prevalence

Other percentages shown were calculated using numbers of service users as denominators, and do not add up to 100% because of multiple providers use.

Table 2 shows the odds ratio of associations with mental health service use for all variables in the full model. The variables that were significantly associated (p < 0.05) with mental health service use, controlling for the presence of other variables, were the same in the final reduced model from backward elimination procedure (Table 3). As expected, the use of mental health services was independently associated with the presence of a diagnosed mental disorder (O.R = 5.27); and additionally with poor or fair self rated mental health (O.R = 2.63 versus excellent or very good) and disability from mental illness (OR = 79.9) (Table 3).

**Table 2:** Logistic regression analyses: full model of determinants of mental health service utilization

		N	Sought treatment					Multivariate					P
				
			N	%	χ^2^	df	P	OR	(95% C.I.)		Wald	df	
Psychiatric disorder:	No	967	32	3.2				1					

	Yes	125	42	33.6	163.7	1	<0.001	5.43	2.29	12.9	14.77	1	<0.001

Disability from mental illness (≥ 1 month)	No	1059	44	4.2									

	Yes	33	30	90.9	381.3	1	<0.001	106.4	24.5	461.9	38.82	1	<0.001

Self-rated mental health:	Excellent or very good	324	14	4.3				1			8.379	2	0.015#

	Good	648	33	5.1				0.82	0.35	1.94	0.198	1	0.657

	Fair of poor	120	27	22.5	52.96	2	<0.001	3.18	1.01	10.0	3.928	1	0.047

Gender	Male	478	26	5.5				1					

	Female	614	48	7.8	0.238	1	0.12	2.25	1.09	4.66	3.386	1	0.07

Age	60–74	824	58	7.0				1					

	≥ 75	268	16	6.0	0.366	1	0.54	0.71	0.31	1.62	0.672	1	0.41

Ethnicity	Chinese	873	61	7.0				1				2	

	Malay	120	6	5.0				0.81	0.25	2.60	0.126	1	0.72

	Indian	99	7	7.1	0.675	2	0.71	0.93	0.30	2.90	0.017	1	0.89

Formal education	No	437	24	5.5				1					

	Yes	655	50	7.5	1.679	1	0.19	2.48	1.11	5.51	4.929	1	0.026

Household income per month	< $1,000	218	22	10.3				1			7.243	2	0.027#

	$1,000 – $2,999	429	33	7.7				0.56	0.25	1.25	2.004	1	0.16

	>$3,000	445	19	4.4	8.607	1	0.015	0.29	0.12	0.72	7.232	1	0.007

Limited by financial resources for medical care	No at all	458	36	7.9				1					

	Yes, to some or great extent	634	38	6.0	1.467	1	0.23	0.38	0.18	0.79	6.791	1	0.009

Actively employed	Yes	164	10	6,1				1					

	No	928	64	6.8	0.004	1	0.85	1.76	0.75	4.15	1.678	1	0.19

Single, divorced, widowed	No	384	34	8.9				1					

	Yes	708	40	5.6	4.047	1	0.044	0.48	0.23	1.03	3.566	1	0.059

Living alone: No	No	1031	69	6.7				1					

Yes	Yes	61	5	8.2	0.206	1	0.65	2.10	0.48	9.22	0.975	1	0.32

Caregiver available	No	44	8	18.2				1					

	Yes	1048	66	6.3	9.440	1	0.002	0.25	0.07	0.78	5.654	1	0.017

Religious or spiritual support	Not at all	169	11	6.5				1			1.819	2	0.40#

	To some extent	504	40	7.7				0.92	0.34	2.50	0.024	1	0.88

	To a great extent	419	23	5.5	1.860	2	0.39	0.57	0.21	1.60	1.123	1	0.29

Regular source of primary care: No	No	163	11	6.8				1					

Yes	Yes	929	63	6.8	0.01	1	1.00	0.66	0.29	1.52	0.941	1	0.33

Prefer to seek professional help: No	No	211	12	5.7				1					

Yes	Yes	881	62	7.0	0.497	1	0.48	0.81	0.34	1.93	0.218	1	0.64

Mental illness can be cured	Not at all	108	3	2.8				1.00			6.866	2	.032#

	To some extent	782	48	6.1				4.85	1.07	22.0	4.178	1	0.041

	To a great extent	202	23	11.4	10.03	2	0.007	8.82	1.69	45.9	6.690	1	0.010

Embarrassed or ashamed about mental illness	Not at all	744	50	6.7				1			0.641	2	0.73

	To some extent	294	19	6.5				1.34	0.63	2.84	0.576	1	0.45

	To a great extent	54	5	9.3	0.576	2	0.75	0.94	0.24	3.66	0.007	1	0.93

Professionals can do little to help	Disagree or strongly disagree	719	36	5.0				1			8.569	2	0.014#

	Neither	246	21	8.5				2.81	1.19	6.65	5.549	1	0.018

	Agree or strongly agree	127	17	13.4	13.55	2	0.001	3.84	1.42	10.4	7.034	1	0.008

Can overcome problem without professional help	Disagree or strongly disagree	549	37	6.6				1			3.096	2	0.21#

	Neither	225	12	5.3				1.57	0.60	4.10	0.851	1	0.36

	Agree or strongly agree	318	25	7.9	1.402	2	0.49	2.02	0.92	4.43	3.072	1	0.08

Drugs often do more harm than good	Disagree or strongly disagree	744	44	5.9				1			2.461	2	0.29#

	Neither	244	24	9.8				1.09	0.47	2.55	0.040	1	0.84

	Agree or strongly agree	104	6	5.8	4.659	2	0.10	0.39	0.11	1.46	1.947	1	0.16

# p for trend

Multivariate model Nagelkerke R^2 ^= 0.510

**Table 3:** Logistic regression analyses: final backward elimination model of determinants of mental health service utilization

		OR	(95% C.I.)		Wald	df	P
Psychiatric disorder:	No	1					

	Yes	5.27	2.40	11.6	17.09	1	<0.001

Disability from mental illness (≥ 1 month)	No	1					

	Yes	79.9	19.3	331.7	36.42	1	<0.001

Self-rated mental health:	Excellent or very good	1			6.997	2	0.030#

	Good	0.84	0.38	1.85	0.183	1	0.67

	Fair of poor	2.63	0.94	7.33	3.408	1	0.065

Gender	Male	1					

	Female	2.106	.953	4.655	4.788	1	0.029

Formal education	No	1					

	Yes	2.40	1.14	5.07	5.219	1	0.021

Household income per month	< $1,000	1			7.396	2	0.026#

	$1,000 – $2,999	0.64	0.30	1.37	1.291	1	0.256

	>$3,000	0.31	0.13	0.72	7.336	1	0.007

Limited by financial resources for medical care	No at all	1					

	Yes, to some or great extent	0.38	0.19	0.76	7.479	1	0.006

Caregiver available	No	1					

	Yes	0.20	0.07	0.59	8.396	1	0.004

Mental illness can be cured	Not at all	1			6.165	2	0.046#

	To some extent	4.01	0.96	16.70	3.649	1	0.056

	To a great extent	6.89	1.47	32.3	5.982	1	0.014

Professionals can do little to help	Disagree or strongly disagree	1			10.91	2	0.004#

	Neither	2.78	1.33	5.81	7.431	1	0.006

	Agree or strongly agree	3.35	1.41	7.97	7.491	1	0.006

# p for trend

Multivariate model Nagelkerke R^2 ^= 0.482

Among non-need variables, female gender (OR = 2.25) and formal education (OR = 2.40) were independently associated with greater mental health service use. Higher household income was associated with less mental health service use (OR = 0.31); a self-report of financial resource limitation for medical care was associated with less mental health service use (OR = 0.38). Reported availability of caregiver was also associated with less use of mental health services (OR = 0.20).

Among health beliefs, a belief that 'mental illnesses can be cured' was significantly associated with more mental health service use ('to a great extent', OR = 6.89; to some extent', OR = 4.01; versus not at all). Paradoxically, believing that 'health professionals can do little to help' was associated with more mental service use (strongly agree or agree, OR = 3.36; neither, OR = 2.78, versus strongly disagree or disagree). Other beliefs were not found to be significantly associated with mental health service use, allowing for the influence of other variables.

## Discussion

In this study, we found as expected that despite the high prevalence of mental health problems, only a minority of seniors sought treatment from health care providers, and if they did, they were most likely to have sought treatment from primary care physicians. We confirmed a number of known determinants of mental health service use, but a number of unique findings shed light on the contributions of personal health beliefs and socio-cultural influences on mental health service use.

As expected, need-for-care clinical and psychiatric factors (diagnosed mental disorder, subjective health status and functional impairment) were primarily associated with the use of mental health services. We also observed similar increased use of mental health service in women in an older population which has been reported in previous studies of the general adult population [19-21]. This is possibly explained in terms of exposure to acute life events, chronic social stresses and lower social status and income and greater self-identification with having a mental health problem and perceived need for help [22]. Our data did not demonstrate that older seniors were more likely to use mental services. Although general adult population studies generally show that the likelihood of treatment for mental illness has been positively linked to older age [6,8,23,24], these findings relate to a wide range of age from younger to older adults, rather than seniors.

The literature reveals a wide discordance in the socio-demographic determinants of need and use of mental health services. Although socioeconomic measures of disadvantage such as unemployment, being unmarried, low income and low education have been shown in many studies [25] to be positively related to the prevalence of psychiatric disorders, their corresponding associations with mental health service use are few and varied. Whereas some general population studies have shown concordant associations of the likelihood of mental illness treatment with being unemployed [8], or being separated, divorced or widowed in some countries [26], we found little of this in our study population. This has also been found to true of most epidemiological studies of populations that differ on demographic characteristics and health care systems. In Finland, for example, where the population has near universal access to services, and the proportion of people using mental health care services is relatively high overall, no sociodemographic factors were found to be associated with mental service use [7].

At the time of the study, Singapore has a health service system that provides equitable and easy access to affordable care over a small geographical area (700 square km). Patients with mental health problems receive affordable primary care treatments from primary care physicians in private or public outpatient clinics or subsidized specialist care in the public psychiatric services (one large psychiatric institute and four general hospital psychiatric units). For the majority (80%) of patients, public hospital care and drug prescriptions for mental illness are partly or fully subsidized according to financial eligibility. Although there appears to be few healthcare delivery barriers to care, we found nevertheless, that a self-report of financial resource limitation for medical care was associated with less mental health service use (OR = 0.38), suggesting that financial barriers to use of mental health services did existed.

In agreement with other studies [21,27,28], we found that seniors with better education were more likely to report treatment for mental illness, suggesting that health literacy in the use of mental health services was closely associated with education. Despite the fact that higher income is associated with better education in Singapore, we found that seniors with higher household income were in fact less likely to use mental health services, contrary to other studies [22]. This may suggest the influence of an unmeasured factor(s) associated with higher income. A possible reason is that households with higher income may be able to afford alternative care arrangement, such as a domestic helper to alleviate the stress of caring for a sick elderly, thus necessitating less medical attention. Indeed, higher income households included significantly more with caregiver support (χ^2 ^= 17.782, 2 df, p < 0.001). However, multivariate analyses indicated that higher income and reported availability of caregiver support were each independently associated with less use of mental services. Together, these resource-associated variables may reflect the presence of an alternative informal social support system, or alternatively, care-giving family members and significant others possibly might hold similar negative beliefs and attitudes as the elderly respondent. Future studies should explore the influence of family member or caregiver resources and attitudes on mental health service use.

Health beliefs are thus an important set of other determinants of mental health services related to health literacy. We found that, importantly, most negative beliefs and attitudes such as embarrassment or shame about mental illness; perceived ability to overcome problem without professional help; perceived harm of drugs showed a lack of association in this population. This may suggest that personally held attitudes did not unfavorably influence seeking help for mental health problems.

Indeed the only association with a negative health belief, 'health professionals can do little to help' was paradoxically associated with more mental service use. This finding appears counter-intuitive but may be explained by the fact that this cross-sectional association reflects the resultant experience of limited or negative outcomes of medical treatment among patients who might have sought treatment from health professionals for more severe mental illnesses.

Positive health beliefs and indices of health literacy could have better predictive utility. This was evidenced by the finding that believing 'mental illnesses can be cured' was strongly associated with more mental health service use, and clearly suggests an important emphasis in health education messages.

## Conclusion

The determinants of mental health service use in the elderly include primary need factors, and the predisposing and enabling factors of female gender and education. There was little evidence of influences by negative personal health beliefs, but clear evidence of the strong influence of a positive health belief that 'mental illness can be cured'. The influence of family members and care-givers on senior's use of mental health service should be further explored.

## Competing interests

The authors declare that they have no competing interests.

## Authors' contributions

MSZN reviewed the literature, and drafted the manuscript. PCC and EHK participated in the design of the study, interpretation of the data, and reviewed the paper; TPN conceived and designed the survey, analyzed and interpreted the data and reviewed the manuscript.
